# 3D Printed Nanocellulose Scaffolds as a Cancer Cell Culture Model System

**DOI:** 10.3390/bioengineering8070097

**Published:** 2021-07-10

**Authors:** Jennifer Rosendahl, Andreas Svanström, Mattias Berglin, Sarunas Petronis, Yalda Bogestål, Patrik Stenlund, Simon Standoft, Anders Ståhlberg, Göran Landberg, Gary Chinga-Carrasco, Joakim Håkansson

**Affiliations:** 1 Unit of Biological Function, Division Materials and Production, RISE Research Institutes of Sweden, Box 857, SE-50115 Borås, Sweden; jennifer.rosendahl@ri.se (J.R.); Mattias.Berglin@ri.se (M.B.); sarunas.petronis@ri.se (S.P.); Yalda.bogestal@ri.se (Y.B.); patrik.stenlund@ri.se (P.S.); Simon.standoft@ri.se (S.S.); 2 Sahlgrenska Center for Cancer Research, Department of Laboratory Medicine, Institute of Biomedicine, Sahlgrenska Academy, University of Gothenburg, Box 425, Medicinaregatan 1G, SE-41390 Gothenburg, Sweden; andreas.svanstroem@gmail.com (A.S.); anders.stahlberg@gu.se (A.S.); goran.landberg@gu.se (G.L.); 3 Wallenberg Centre for Molecular and Translational Medicine, University of Gothenburg, SE-40530 Gothenburg, Sweden; 4 Department of Clinical Genetics and Genomics, Region Västra Götaland, Sahlgrenska University Hospital, SE-40530 Gothenburg, Sweden; 5 Department of Clinical Pathology, Sahlgrenska University Hospital, SE-41345 Gothenburg, Sweden; 6 RISE PFI AS, Høgskoleringen 6b, NO-7491 Trondheim, Norway; 7 Department of Laboratory Medicine, Institute of Biomedicine, University of Gothenburg, P.O. Box 440, SE-40530 Gothenburg, Sweden

**Keywords:** nanocellulose, 3D printing, cancer, 3D cell culture, CNF, cancer stemness

## Abstract

Current conventional cancer drug screening models based on two-dimensional (2D) cell culture have several flaws and there is a large need of more in vivo mimicking preclinical drug screening platforms. The microenvironment is crucial for the cells to adapt relevant in vivo characteristics and here we introduce a new cell culture system based on three-dimensional (3D) printed scaffolds using cellulose nanofibrils (CNF) pre-treated with 2,2,6,6-tetramethylpyperidine-1-oxyl (TEMPO) as the structural material component. Breast cancer cell lines, MCF7 and MDA-MB-231, were cultured in 3D TEMPO-CNF scaffolds and were shown by scanning electron microscopy (SEM) and histochemistry to grow in multiple layers as a heterogenous cell population with different morphologies, contrasting 2D cultured mono-layered cells with a morphologically homogenous cell population. Gene expression analysis demonstrated that 3D TEMPO-CNF scaffolds induced elevation of the stemness marker *CD44* and the migration markers *VIM* and *SNAI1* in MCF7 cells relative to 2D control. T47D cells confirmed the increased level of the stemness marker *CD44* and migration marker *VIM* which was further supported by increased capacity of holoclone formation for 3D cultured cells. Therefore, TEMPO-CNF was shown to represent a promising material for 3D cell culture model systems for cancer cell applications such as drug screening.

## 1. Introduction

Cancer is one of the most abundant diseases worldwide and there is a clear need for developing new and effective drugs and therapies [1]. Such development requires relevant pre-clinical in vitro models that adequately represent the human situation. Conventional cell cultures in plastics have been applied to fill this gap [2]; however, in most situations these cell cultures do not represent a relevant tissue mimicking its natural environment and consequently only 5% of new drug candidates that enter clinical trial reach the market with a proper safety profile and shown effect in humans [3,4,5]. Hence, the scientific community needs improved tumor model systems.

There is increasing evidence that cancer stem cells (CSC) which possess the capacity for self-renewal, unlimited proliferation and multidrug- and radiotherapy resistance are the driving force in many cancer types [6,7]. Two-dimensional (2D) cell cultures are usually grown in a mono layer and have a homogenous cell population with limited CSC properties. Three-dimensional models represent the in vivo situation more closely by inducing the cells to grow in multiple layers, stimulate CSC properties, communicate in all directions and enable representative cellular heterogeneity [2,8,9]. Three-dimensional culturing platforms represent promising predictive models for in vivo tumorigenesis [9,10,11] as the microenvironment, both structurally, biochemically and biomechanically, plays a vital role in cell fate, differentiation, organ development, signal transduction and countless other biological processes [12,13]. A cell culture tumor model system would therefore benefit from a microenvironment that stimulates the formation of a heterogeneous cell population with CSC traits in order to represent a physiological tumor microenvironment.

Various biopolymers have been applied for 3D cell culture including polysaccharides and proteins, e.g., fibrin, alginate and collagen [14,15]. These have been reported to be biocompatible and to alter cellular response [16], which is relevant for mimicking complex tissues. Preclinical cell culture models should be able to mimic the tumor microenvironment and may also benefit from physiologically non-biodegradable structures in order to provide support and stability to the cell cultures. In addition, the ability to 3D print biopolymers is beneficial for constructing optimized 3D models based on architectural preference. Wood nanocellulose has proven to be a suitable biobased material for various biomedical applications. Cellulose nanofibrils (CNF) has been extensively studied as gels for wound healing, drug carriers and scaffolds for tissue engineering [17,18,19,20,21]. This is due to the versatility of CNF grades regarding properties such as biocompatibility and the capability to form gels at low concentration with adequate viscoelastic behavior, the latter being most interesting from a 3D bioprinting perspective [22]. Various types of CNF grades have an appropriate viscosity at low concentrations and good shear thinning behavior [23,24,25]. The gels can easily be extruded through a nozzle, deposited on a substrate and maintain a defined 3D shape post printing due to the rapid zero-shear viscosity recovery [26,27,28,29]. Carboxylated CNF, produced through 2,2,6,6-tetramethylpiperidinyl-1-oxyl (TEMPO)-mediated oxidation [30], has been used in various studies concerning biomedical applications demonstrating benefits for cell viability, proliferation, migration, biocompatibility and cytotoxicity [18,19]. In addition, TEMPO-CNF has been shown to have a positive effect on fibroblast, HeLA and Jurkat tumor cell growth and proliferation [31], indicating the potential of TEMPO-CNF as a printing ink for 3D cell culturing applications.

The aim of this study was to evaluate TEMPO-CNF as a 3D printable ink to form scaffolds for tumor cell culturing and to study the effect of the 3D model on cell behavior. We demonstrate how 3D printed TEMPO-CNF may induce cancer stemness and affect cellular morphology and heterogeneity, which suggest TEMPO-CNF to be a promising material for cell culturing in 3D.

## 2. Materials and Methods

### 2.1. Nanocellulose

Bleached *Pinus radiata* kraft pulp fibers were kindly provided by CMPC (Planta Pacifico, Chile). The chemical composition of the pulp fibers has been quantified to 87% cellulose, 12.2% hemicellulose and 0.8% lignin [32]. The pulp fibers were chemically pre-treated with 2,2,6,6-tetramethylpiperidinyl-1-oxyl (TEMPO) mediated oxidation [33], applying 3.0 mmol/g hypochlorite (NaClO, 9%) with the reaction kept at pH 10. The pulp fibers were washed with deionized water to achieve a conductivity of less than 5 μS/cm to secure removal of unreacted reactants. The pre-treated pulp fibers in deionized water (1% *w*/*v*) were homogenized using a Rannie 15 type 12.56X homogenizer operating at 1000 bar pressure. Conductometric titration was applied to measure the carboxylic acid content of the produced TEMPO-CNF and was quantified to 982 ± 8 µmol/g [34]. The nanocellulose was stored at 2–8 °C until 3D-printing.

### 2.2. Atomic Force Microscopy and Nano-Mechanical Assessment

Atomic force microscopy analysis (AFM) was performed on the TEMPO-CNF to reveal the morphology of single cellulose nanofibrils. AFM (Veeco multimode) was performed using tips with a spring constant of ∼0.4 N m^−1^ (Bruker AFM probes) on areas of 2 × 2 µm (1024 × 1024 pixels). The stiffness of the scaffolds was analyzed using nano-mechanical assessment. The freeze-dried samples were immersed in either DMEM- or RPMI-medium (Thermo Fisher Scientific, Gothenburg, Sweden) for 1 h prior to measurement and the following nanoindentation parameters were applied: Berkovich tip; displacement controlled at peak indentation depth of 2000 nm; 0.125 s loading, 0.4 s holding, 0.125 s unloading (total testing time 0.65 s for one indent). More than 20 indents on random areas on each sample were performed and two replicate samples were assessed in solution.

### 2.3. 3D Printing

TEMPO-CNF hydrogel with a concentration of 1% (*w*/*v*) in water was used as ink for 3D printing. Previously, viscosity data of the same CNF gel and the corresponding printability was reported [35]. In the present study, the CNF was 3D printed in a laminar airflow hood as square box scaffolds with the dimension 30 × 30 × 5 mm; grid distance 1.5 mm, 90°, with an outer frame in 4 layers to produce a mesh like scaffold (3DPS) using an EnvisionTEC 4th Gen 3D-Bioplotter (EnvisionTEC, Gladbeck, Germany). The 3D print was designed using CAD (computer aided design) software, Autodesk Fusion 360 2019 (Autodesk Inc., San Rafael, CA, USA) and exported to the printer as an STL-file. The printing parameters for TEMPO-CNF were: temperature (20 °C), pressure (0.2 bar), speed (70 mm/s) and needle offset (0.50 mm). The printing was controlled using Visual machine software (EnvisonTEC, Gladbeck, Germany) and a tapered needle with 410 µm internal diameter. The strand width of the printed nanocellulose was measured on images acquired directly after printing with the bioprinter EnvisionTEC 4th Gen 3D-Bioplotter (EnvisionTEC, Gladbeck, Germany) using ImageJ software (1.52a). The data was plotted in GraphPad Prism v9 (GraphPad). The scaffolds were frozen at −20 °C, transferred to −80 °C and then freeze-dried for at least 48 h, or to stable weight, using VirTis Sentry 2.0 Benchtop Freeze Dryer (SP Scientific, Warminster, PA, USA). Each scaffold was cut into 9 pieces using a scalpel.

### 2.4. 2D Cell Culture

Conventional polystyrene culture plates were used for 2D cell culture (6-well plate, Sarstedt, Helsingborg, Sweden). The breast cancer cell line MCF7 (ATCC) was cultured in 1X DMEM (Thermo Fisher Scientific, Gothenburg, Sweden) supplemented with 10% *v*/*v* fetal bovine serum (Thermo Fisher Scientific, Gothenburg, Sweden), 1% *v*/*v* L-glutamine (Thermo Fisher Scientific, Gothenburg, Sweden), 1% *v*/*v* penicillin/streptomycin (Thermo Fisher Scientific, Gothenburg, Sweden) and 1% *v*/*v* MEM non-essential amino acid solution (100X) (Sigma-Aldrich, Stockholm, Sweden). The breast cancer cell lines MDA-MB-231 (ATCC) and T47D (ATCC) were cultured in 1X RPMI (Thermo Fisher Scientific, Gothenburg, Sweden) supplemented with 10% *v*/*v* FBS, 1% *v*/*v* L-glutamine, 1% *v*/*v* penicillin/streptomycin and 1% *v*/*v* 100 mM sodium pyruvate (Thermo Fisher Scientific, Gothenburg, Sweden). Cells were seeded with a density of 2.0–2.5 × 10^5^ cells per well in 6-well plates and cultured for 48 h for holoclone formation and gene expression analysis, respectively, or 1 × 10^5^ cells per well in 6-well plates for up to 8 d for cell growth assessment. Longer culturing time was not used to avoid confluence and cell arrest, which would otherwise make the cells incomparable with growth in 3D-printed scaffolds. All cell lines were cultured at 5% CO_2_ at 37 °C.

### 2.5. 3D Cell Culture

To grow cells in 3D printed scaffolds, cells were detached from 2D culture plates using 0.25% Trypsin-EDTA (Thermo Fisher Scientific, Gothenburg, Sweden), centrifuged at 300× *g* for 3 min and the cell pellet was resuspended in cell medium. The freeze-dried scaffolds were pre-wetted in in cell culture medium for 15 min before adding cells in desired density on top of the scaffolds. Cells were counted using MOXI, (Orlflo, Ketchum, ID, USA), seeded with a density of 3 × 10^5^ cells (for cell viability assay) or 5 × 10^4^ cells (for quantitative PCR (qPCR) and scanning electron microscopy analysis) per well in a 48 well plate. Three-dimensional prints were transferred to 6-well plates (Polystyrene, Thermo Fisher Scientific, Gothenburg, Sweden) after 24 h and thereafter transferred to new medium every 3–4 d until final analysis. Gene expression analysis with qPCR and imaging with SEM was performed after 14 d, and holoclone formation was analyzed after 21 d of culture.

### 2.6. Cell Viability Assessment

Cells were detached as described above, centrifuged at 300× *g* for 3 min and resuspended in cell medium and trypan blue 1:2 (Thermo Fisher Scientific, Gothenburg, Sweden). The number of live and dead cells were counted using a dispensable hemocytometer (C-Chip, NanoEnTek, Seoul, Korea).

### 2.7. Gene Expression Analysis

Scaffolds were gently washed twice in PBS, transferred to 2 mL tubes, lysed with 600 µL QIAzol lysing reagent (Qiagen, Kista, Sweden) and 50 µL lysis buffer (5 µL RNase out (Thermo scientific, Gothenburg, Sweden), 2.5 µL BSA 20 µg/µL (Thermo scientific, Gothenburg, Sweden) and 42.5 µL RNase free water), snap-frozen in liquid nitrogen and stored at −80 °C. The samples were thawed on ice for 30 min and disrupted using 5 mm stainless steel beads (Qiagen, Kista, Sweden) and TissueLyser II (Qiagen, Kista, Sweden) for 2 × 2.5 min at 25 Hz. Total RNA isolation was extracted using miRNeasy Micro kit (Qiagen, Kista, Sweden) with on-column DNase digestion according to the manufacturer’s instructions and RNA concentration was determined using a NanoDrop (ND-1000, Saveen Werner AB, Malmo, Sweden). Complementary DNA (cDNA) was produced using the GrandScript cDNA synthesis kit (TATAA Biocenter, Gothenburg, Sweden) and a T100 Thermal Cycler (Bio-Rad, Solna, Sweden). Reverse transcription was performed in 10 µL reactions at 25 °C for 5 min, 42 °C for 30 min and 85 °C for 5 min followed by cooling to 4 °C. The cDNA was diluted 1:10 in RNase free water (Invitrogen) and pre-amplified in 50 µL reactions with 1 × SYBR GrandMaster Mix (TATAA Biocenter, Gothenburg, Sweden), 0.04 µM of each primer in a primerpool (Supplementary Table S1) and 1 µg/mL of BSA (Thermo Scientific, Gothenburg, Sweden) using a CFX96 Touch Real Time Cycler (Bio-Rad) and the following temperature profile: 95 °C for 3 min, 15 cycles at 95 °C for 20 s, 60 °C for 3 min and 72 °C for 20 s. The samples were snap frozen on dry ice, thawed on ice and diluted 20 times in TE-buffer (Thermo scientific, Gothenburg, Sweden). For qPCR, all samples were diluted 1:3 and run in 6 µL reactions with 400 nM primers (Supplementary Table S1) and 1 × SYBR GrandMaster Mix (TATAA Biocenter, Gothenburg, Sweden). qPCR was run using a CFX384 Touch Real Time Cycler (Bio-Rad, Solna, Stockholm) with the following temperature profile: 95 °C for 2 min, 39 cycles at 95 °C for 5 s, 60 °C for 20 s and 70 °C for 20 s followed by a melting curve analysis at 65 °C to 95 °C with 0.5 °C per 5 s increments. CFX Manager Software version 3.1 (Bio-Rad, Solna, Stockholm) regression method was used to determine the cycles of quantification (Cq) values. Data was analyzed using GenEx software (MultiD). Samples with >25% missing values were removed followed by the same criteria for genes. Missing values were imputed based on replicates, cut-off and missing Cq-values were set to 35. Data was normalized to reference genes identified with NormFinder algorithm, transformed to relative values and log2 scale. For each experiment optimal reference genes were evaluated. Minimum information for publication of quantitative real-time PCR experiments guidelines was followed for qPCR [36].

### 2.8. Scanning Electron Microscopy (SEM)

Cells cultured for 48 h in 2D culture plates, or for 14 d in 3DPS, were gently washed once in cell medium and fixated for 1 h at room temperature in 2.5% glutaraldehyde (Product ref G7651-10ML, Sigma-Aldrich Sweden AB, Stockholm, Sweden) diluted in cell medium. Scaffolds were washed once in TBS (50 mM Trizma-HCl, Sigma-Aldrich, Stockholm, Sweden; 150 mM NaCl, Merck, Stockholm, Sweden; pH 7.5) with 0.1 mM CaCl_2_ (Merck, Stockholm, Sweden), fixated for 1 h at room temperature in 1% osmium tetroxide (Sigma-Aldrich, Stockholm, Sweden) in TBS (50 mM Trizma-HCl (Sigma-Aldrich, Stockholm, Sweden); 150 mM NaCl, (Merck, Stockholm, Sweden); pH 7.5) with 0.1 mM CaCl_2_ (Merck, Stockholm, Sweden), rinsed in deionized water, plunge-frozen in liquid propane (EMS-002 Rapid Immersion Freezer, Electron Microscopy Sciences) and freeze-dried overnight (VirTis Sentry 2.0 Benchtop Freeze Dryer, SP Scientific, Warminster, PA, USA). Samples were mounted on aluminum stubs by adhesive carbon tabs (Agar Scientific) and coated with a 10 nm Au/Pd conducting thin film by sputter-coater (PECS Mod 682, Gatan Inc., Pleasanton, CA, USA). Samples were imaged by Zeiss SUPRA 40VP scanning electron microscope operated in secondary electron mode at 3.0–4.1 kV acceleration voltage, 9–17 mm working distance and 100–250,000× magnification range.

### 2.9. Hematoxylin and Eosin Staining

TEMPO-CNF scaffolds with cells cultured for 14 d were gently washed once in cell medium and fixated for 1 h at room temperature in 4% formaldehyde (Histolab, Gothenburg, Sweden) diluted in PBS (Medicago, Uppsala, Sweden). The formaldehyde liquid was removed and replaced with 70% ethanol for 1 h at room temperature, followed by 95% ethanol for 1 h at room temperature, 99% ethanol for 1 h at room temperature and finally xylene (Univar) for 1 h at room temperature. Scaffolds were paraffin embedded, sectioned to 4.5 µm thickness using a retracting microtome (Rotary one, LKB Stockholm, Sweden), dried overnight at 60 °C, de-paraffinized, counterstained with hematoxylin and eosin, de-hydrated and mounted with pertex (Histolab, Gothenburg, Sweden). Sections were imaged with Leica SCN400 Slide Scanner (Meyer instruments, Houston, TX, USA). Representative images were selected and exported using Leica SlidePath Gateway software and analyzed with ImageJ software [37].

### 2.10. Holoclone Assay

Cells cultured for 48 h in 2D or for 14 d on TEMPO-CNF were detached as described above and seeded in 6-well plates at a density of 50 cells/cm^2^, cultured for 6–7 d to form colonies and stained with crystal violet. Holoclones were identified as colonies (≥32 cells) with small clustered as well as differentiated cells and quantified using Gelcount software (Oxford Optronix, Oxford, UK).

### 2.11. Statistics

All statistical data analyses were performed using GraphPad Prism v8 (GraphPad). The values in the graphs are presented as average ± standard error of the mean.

## 3. Results

### 3.1. Nanocellulose Characteristics

**Figure 1:**
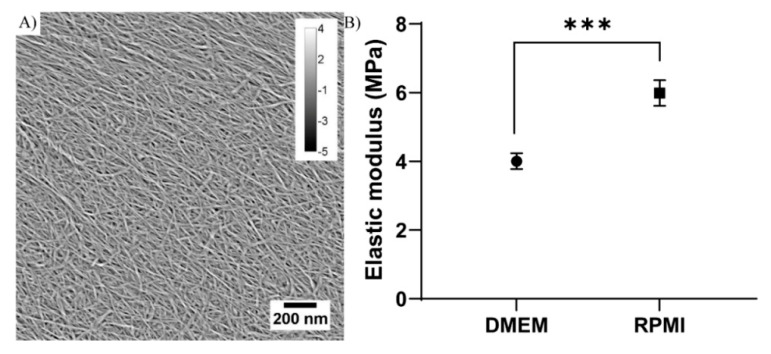
Structural morphology and stiffness of TEMPOCNF. (**A**) AFM nanoscale image of nanofibrils with diameter <20 nm. The scale bar (lower right) and calibration (upper right) indicate the scales in nanometers, in the x, y and z-directions, respectively. (**B**) Elastic modulus of analysis on TEMPO-CNF in DMEM and RPMI medium, respectively. The dots represent average elastic modulus and error bars represent standard error of the mean (*n* = 42–43); unpaired *t*-test. *** *p*-value < 0.001.

In order to develop an in vivo-like 3D cell culture system to be used as a cancer drug screening model, TEMPO-CNF was used as a base material to produce extrusion 3D printed scaffolds. After 3D printing, the TEMPO-CNF colloidally stable gel material was transformed to a porous TEMPO-CNF structure by a freeze-drying procedure and the nanomorphology confirmed the formation of close-packed nanofibrillar networks (nanofibril diameter <20 nm) (Figure 1A). Mechanical properties of the TEMPO-CNF hydrogel were analyzed with nanoindentation, and the quantified elastic modulus was comparable with previously published results, i.e., lower MPa regime [15], but varied with growth medium. Different cells lines need different culture condition and in the present study, DMEM and RPMI cell growth medium was used. It was found that the different cell growth media affected the elastic modulus of the material where RPMI statistically significantly increased the material stiffness with 50% compared with DMEM with elastic modulus’ of approximately 4 MPa for DMEM and 6 MPa for RPMI (Figure 1B).

### 3.2. Printability Evaluation

**Figure 2:**
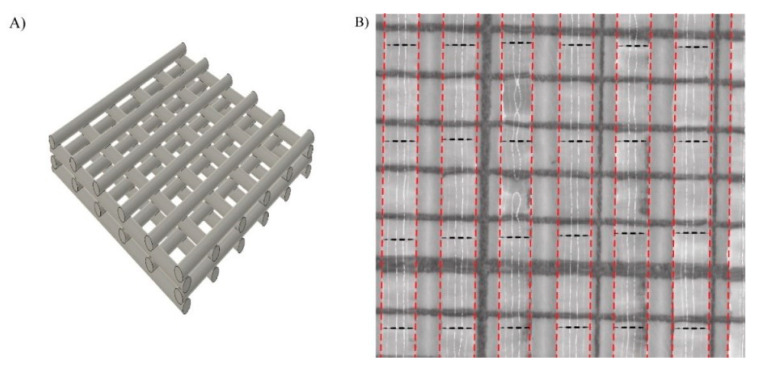
Design of scaffold and printability of TEMPO-CNF (**A**) Schematic illustration of the printed grid model. (**B**) One layer of printed TEMPO-CNF strands on mm-marked paper (black solid lines in the image) to determine the strand width. The strands are marked with red dotted lines and the measurement position is marked with black dotted lines.

Nanocellulose was printed in a 4-layer grid structure to create a porous mesh in which the cells and nutrients could access the whole scaffold (Figure 2A). The printability of the TEMPO-CNF material was evaluated by analyzing the flow out of the printed strands. This was performed by determining the strand width directly after printing, and was assessed to 0.86 ± 0.07 mm (average ± standard error of the mean) when printed with a 410 µm needle (Figure 2B), which is similar to other materials, e.g., alginate and gelatin mix [38]. The average pore size between strands was 0.52 ± 0.01 mm^2^ (average ± standard error of the mean) and the pore size within the material ranged from <1 × 10^−6^ to 0.2 mm^2^. The strand and pore size of the 3D printed construct were similar to previous studies where the same CNF ink was used [35].

### 3.3. 3D Printed CNF Suitable as Cell Growth Scaffolds

**Figure 3:**
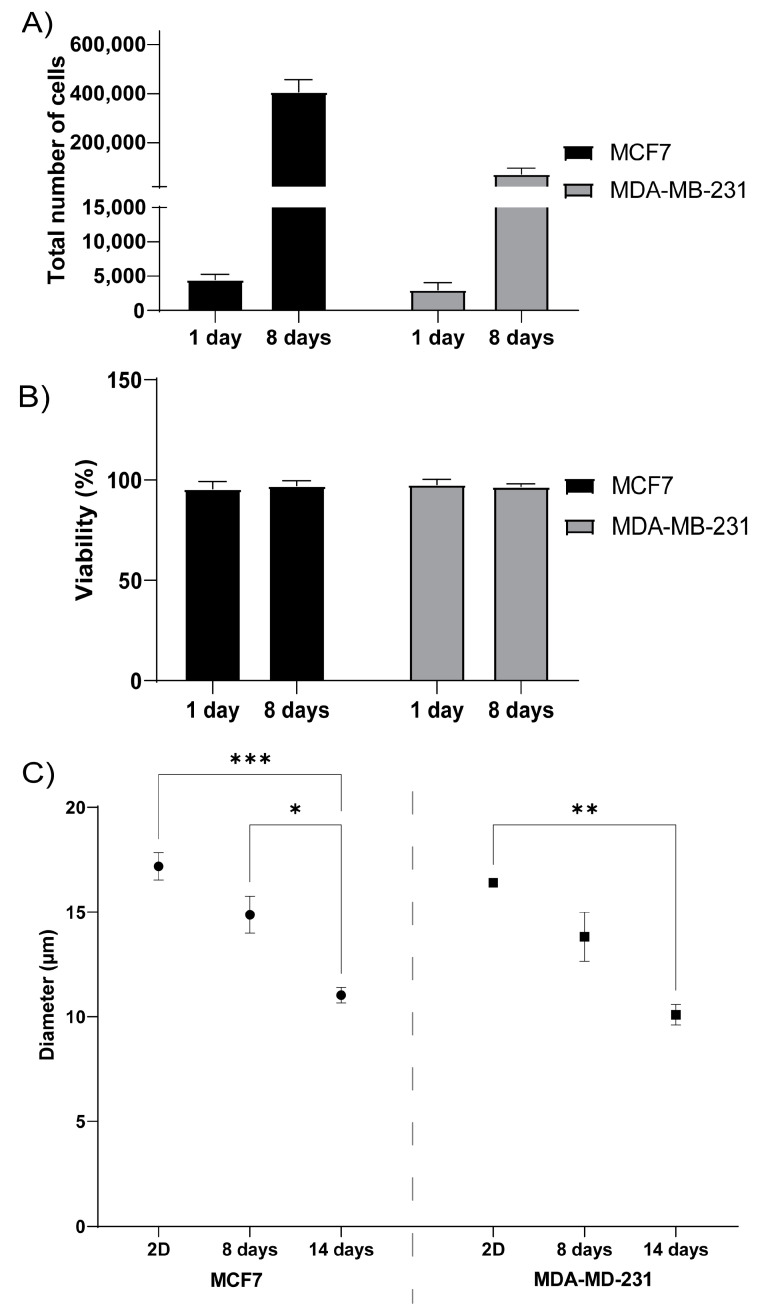
3D printed TEMPO-CNF as a suitable scaffold for breast cancer cells. (**A**) Total number of MCF7 and MDA-MB-231 breast cancer cells quantified after 1- and 8-days culture, respectively, in 3D printed TEMPO-CNF scaffolds, *n* = 6–9. (**B**) Viability of MCF7 and MDA-MB-231 cells quantified after 1- and 8-days culture, respectively, in 3D printed TEMP-CNF scaffolds, *n* = 6–9. (**C**) Quantification of cell diameter of MCF7 and MDA-MB-231 cells cultured in 2D or in 3D printed TEMPO-CNF scaffolds for 8 and 14 days, respectively, *n* = 3–9. (**A**–**C**) Average ± standard error of the mean. Two-way ANOVA; Sidak’s post hoc test for multiple comparison. * *p*-value < 0.05, ** *p*-value < 0.01, *** *p*-value < 0.001.

To evaluate how well cells grow in 3D printed TEMPO-CNF structures, the estrogen receptor- and progesterone receptor positive breast cancer cell line MCF7 or the estrogen receptor- and progesterone negative breast cancer cell line MDA-MB-231 were seeded on the scaffolds. Both cell lines attached to the scaffolds to the same extent, as analyzed by the cell number after 1 day of culture, and expanded well during the following 7 days, approximately 90-fold for MCF7 and 24-fold for MDA-MB-231 (Figure 3A). The cell viability was assessed to >96% for both cell lines, at both 1 and 8 days of culture, which further confirmed the material and growth conditions as suitable (Figure 3B). By comparing the cell size between cells grown in 2D cell culture dishes with 3D printed TEMPO-CNF scaffolds, it was shown that both MCF7 and MDA-MB-231 cells were significantly smaller in 3D compared with 2D after 14 days culture, 36% and 38%, respectively. Interestingly, the cell size also decreased from 8 to 14 days culture in the 3D environment, 24% and 30% for MCF7 and MDA-MB231, respectively (Figure 3C).

**Figure 4:**
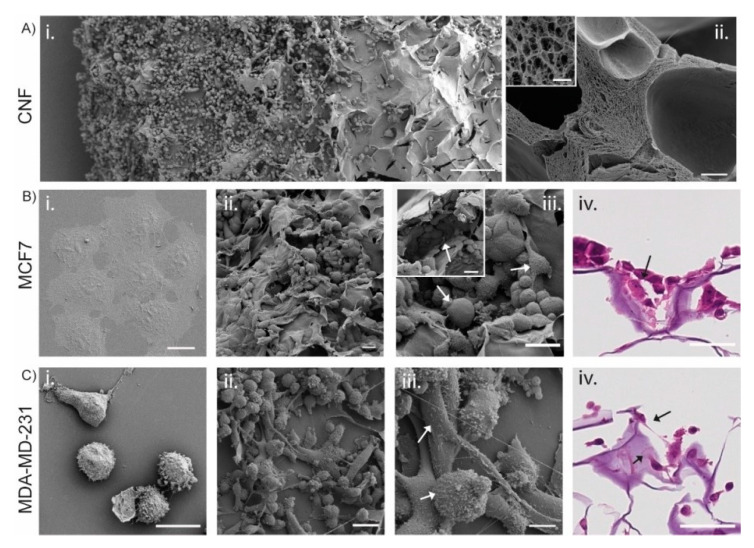
Cells cultured on TEMPO-CNF develop a heterogenous cell population with different cell morphologies. (**A**,**B**(**i**–**iii**),**C**(**i**–**iii**)) SEM images of (**A**) TEMPO-CNF material in (**i**) low magnification overview with MDA-MB-231 cells and (**ii**) cross section and cavities in the material. Inset in (**A**(**ii**)) higher magnification of the cross section. (**B**) MCF7 and (**C**) MDA-MB-231 cells. (**B**(**i**),**C**(**i**)) Cells grown in 2D cell culture plates in a homogenous mono layer. (**B**(**ii**,**iii**),**C**(**ii**,**iii**)) illustrates cells grown in multiple layers with different morphologies (indicated by white arrows in (**B**(**iii**),**C**(**iii**)). Inset in Biii shows cells filling out holes and cavities in the TEMPO-CNF material. (**B**(**iv**),**C**(**iv**)) Hematoxylin and eosin-stained sections of TEMPO-CNF scaffolds with MCF7 and MDA-MB-231, respectively. Black arrows indicate the cells. Scale bars 200 µm (**A**(**i**)), 20 µm (**B**(**i**),**C**(**i**)), 10 µm (**A**(**ii**),**C**(**iii**)), 1 µm (inset in (**A**(**ii**))), 10 µm (**C**(**iv**)), 30 µm ((**B**(**ii**–**iv**),**C**(**ii**)), inset (**B**(**iii**))).

To analyze the microscopic structure of the 3D printed TEMPO-CNF and evaluate the scaffolds’ ability to affect the cellular phenotype, MCF7 and MDA-MB-231 cells were analyzed with SEM and histochemistry after growth on TEMPO-CNF scaffolds for 14 days or in conventional 2D cell cultured for 2 days (Figure 4). The surface of the 3D printed TEMPO-CNF material displayed a porous structure at two distinct scales. Elongated large pores with cavity sizes up to several hundred microns with a sheet-like and rather smooth wall surface for cells to attach to (Figure 4A(i)). In higher magnification, we could see TEMPO-CNF networks forming open pores of sub-micron size enabling cell medium transport (Figure 4A(ii)). In 2D culture, the cells grew in a mono layer as morphologically homogenous populations, (Figure 4B(i),C(i)). In contrast, cells cultured in 3D printed TEMPO-CNF scaffolds grew in multiple layers and developed cellular heterogeneity. This was shown by different morphologies in association with pores and cavities illustrated by low and high magnification images with SEM (Figure 4B(ii,iii),C(ii,iii)) and hematoxylin and eosin-stained paraffin sections (Figure 4B(iv),C(iv)).

### 3.4. 3D CNF Environment Induce Stem Cell Properties

**Figure 5:**
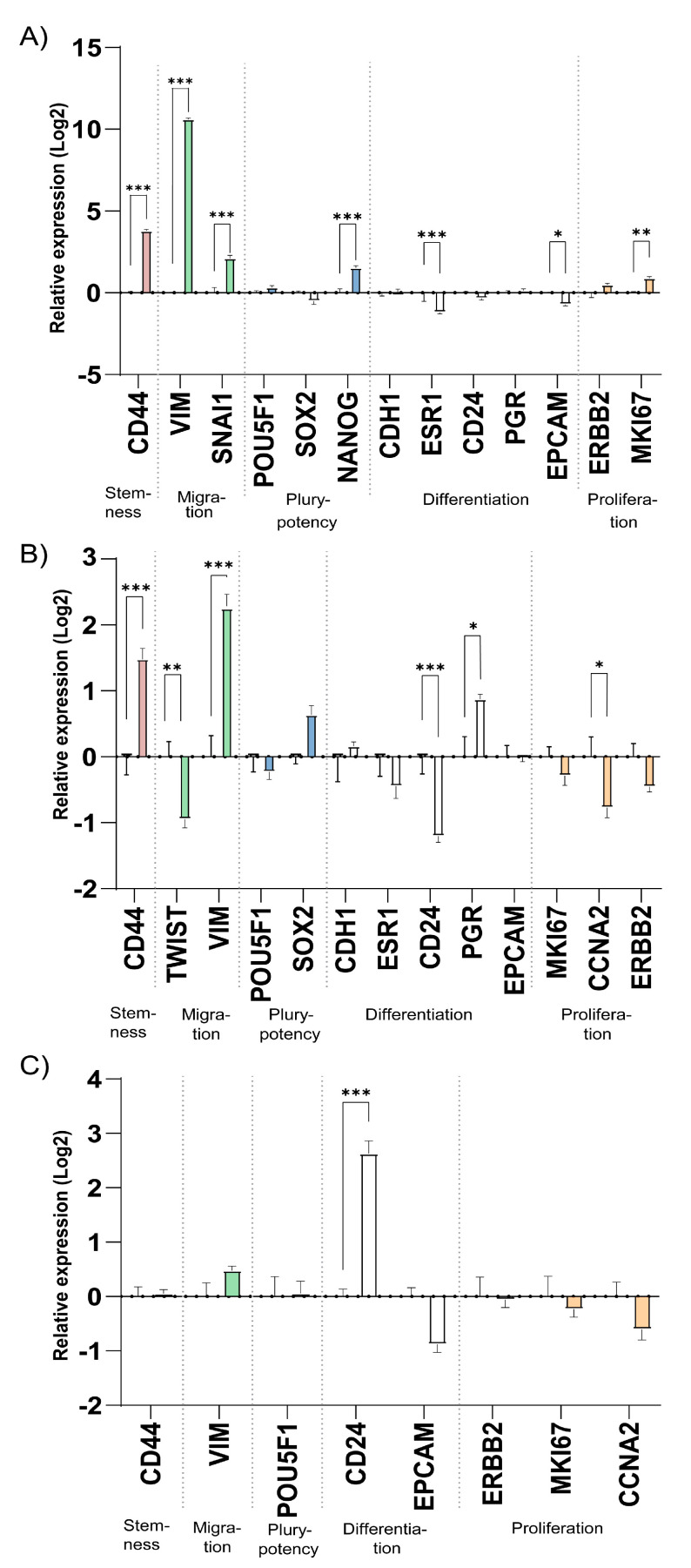
The microenvironment of 3D printed TEMPO-CNF scaffolds alters expression of stemness and migration gene markers. Gene expression levels analyzed by qPCR on cells cultured for 2 weeks in 3D scaffolds and 48 h in 2D for the cell lines (**A**) MCF7, (**B**) T47D and (**C**) MDA-MB-231. The data was normalized against the gene expression of the 2D control, set to zero for each gene. Average ± standard error of the mean (*n* = 3). Two-way ANOVA; Sidak’s post hoc test for multiple comparison (2D). * *p*-value < 0.05, ** *p*-value < 0.01, *** *p*-value < 0.001.

Cells adjust their gene expression profile depending on the microenvironment and growth conditions. Here, we profiled genes related to stemness, migration and epithelial-mesenchymal transition (EMT), pluripotency, differentiation and proliferation in cells cultured in 3D printed TEMPO-CNF scaffolds compared with 2D plastic cell culture. In addition to MCF7, another estrogen receptor- and progesterone receptor positive breast cancer cell line, T47D, was used for gene expression studies. Relative to the expression in 2D cell culture, the 3D TEMPO-CNF environment induced expression of the stemness marker *CD44* and the migration marker *VIM* in both cell lines. The migration marker *SNAI1* was upregulated in MCF7 while *TWIST* was downregulated in T47D. The pluripotency marker *NANOG* was slightly upregulated in MCF7. *ESR1* and *EPCAM* were downregulated in MCF7, and *CD24* and *PGR* was down- and upregulated in T47D, respectively. The proliferation marker *MKI67* was slightly upregulated in MCF7, while *CCNA2* was more downregulated in T47D. In the estrogen receptor- and progesterone negative breast cancer cell line MDA-MB-231, the only significantly detected gene expression change was upregulation of the differentiation marker *CD24*, (Figure 5).

**Figure 6:**
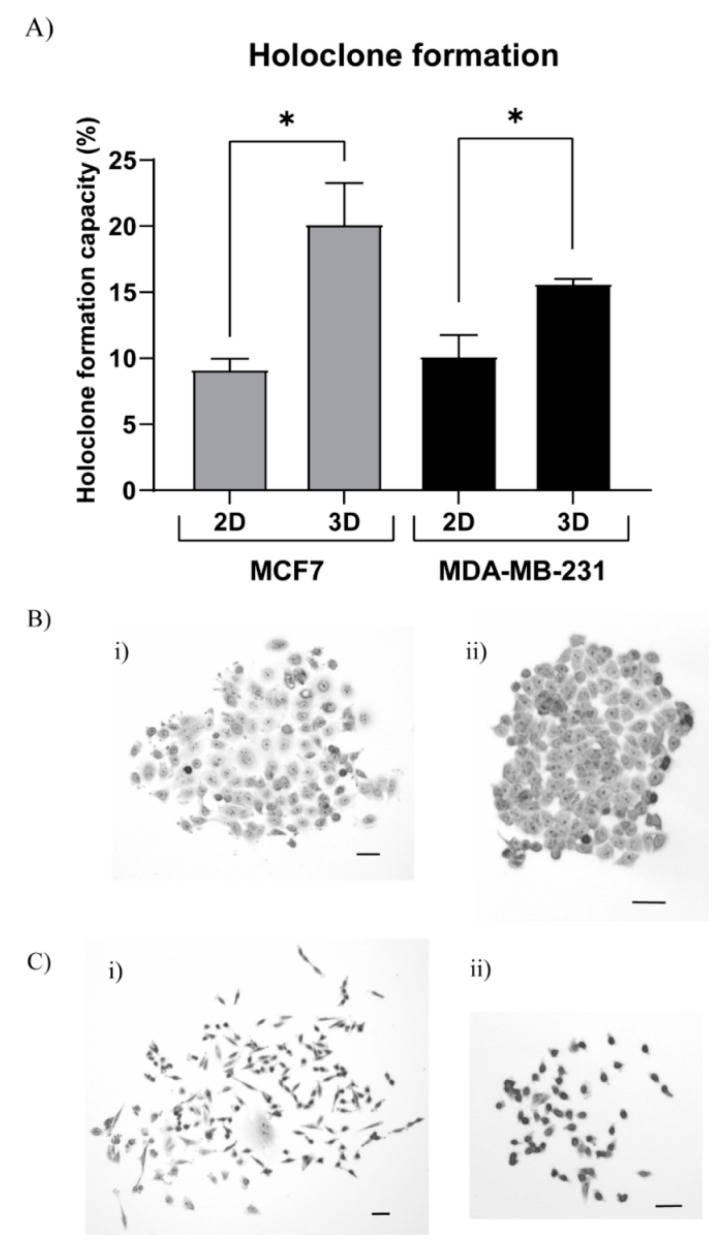
3D TEMPO-CNF environments increase holoclone formation properties. MCF7 and MDA-MB-231 cells were cultured in 3DPS and 2D for 2 weeks or 48 h, respectively, detached and analyzed for holoclone formation properties. (**A**) The holoclone formation ability was quantified for MCF7 and MDA-MB-231 cells grown in 3D TEMPO-CNF scaffolds compared with 2D culture. Unpaired *t*-test: Gaussian distribution between 2D and 3D within each cell line. Average ± standard error of the mean, * *p*-value < 0.05, *n* = 9. Holoclone formation assay with (**B**) MCF7 and (**C**) MDA-MB-231 cells grown in 3D TEMPO-CNF scaffolds. Images illustrates differentiated cells (**B**(**i**),**C**(**i**)) and holoclones (**B**(**ii**),**C**(**ii**)). Scale bars 50 µm.

To test the stemness properties of the 3D TEMPO-CNF cultured MCF7 and MDA-MB-231 cells at cellular level we performed the holoclone formation assay. The 3D TEMPO-CNF environment significantly increased the ability to form holoclones for both MCF7, with 120%, and MDA-MB-231 cells, with 54%, compared with 2D cultured cells (Figure 6A).

## 4. Discussion

In attempts to mimic native human tissue, there are several ways to produce scaffolds as a structural base for tissue engineering or in vitro 3D cell culturing models. For 3D printing applications, an extensive material database for bio-inks has been developed during the last decade [39] and the various materials have different limitations and unique properties depending on how the material is alternated, what cell types to be used and the application strategy. One promising option is the various types of nanocelluloses that can be obtained from different raw materials, including wood, of which hard- and softwood chemical pulp fibers are the most commonly used to produce nanocelluloses. Wood pulp fibers are treated chemically or enzymatically, to generate two main types of wood nanocellulose fibers, i.e., CNF and cellulose nanocrystals. In this work, we present a novel approach where TEMPO-CNF [34,40] is 3D printed as scaffolds for cancer cells to develop a 3D cell growth system simulating the human cancer environment. To be able to obtain a good understanding of how cells interact with the material, it is important to perform a thorough material characterization. The chemical modifications on the TEMPO-CNF used in this study introduced carboxylic functionality on the surface of the material. Hence, the interaction with cells could, in addition to hydrogen bonds, act via electrostatic interaction as well as cationic metal chelation. The carboxyl acid content of the TEMPO-CNF was 982 ± 8 µmol/g [34] and similar surface charge of TEMPO-CNF has previously been demonstrated to be suitable for cell growth and proliferation [34,41].

The mechanical properties of the material also have a critical role regulating the cell phenotype and function [42]. The range of elastic modulus for cancer tissue is wide and has generally been found to vary from 800 to 4500 Pa depending on location [43]. However, in calcified regions the elastic modulus can be much higher locally and reach the same as bone. So, even if the TEMPO-CNF hydrogels are considerably stiffer than cancer tissue, they could, from a cellular perspective, belong to the same microenvironmental niche and the biological response can be comparable. The response to elastic modulus could hypothetically be studied by varying the TEMPO-CNF-concentration during the preparation of the TEMPO-CNF hydrogel and should not vary the porosity and nanostructure.

When TEMPO-CNF is dispersed in water to form a gel, the individual nanofibrils interact with each other and, at a sufficient concentration, establishes a percolated network thus increasing the corresponding viscosity. The concentration of nanofibrils used in this study is above the concentration to form network percolation reported for TEMPO-CNF [23]. The TEMPO-CNF at 1% therefore has suitable viscosity for 3D printing [35] as demonstrated in the present study (Figure 2).

Depending on the composition of the cell culture medium used, the material will behave differently, e.g., cations will react with carboxyl groups in the TEMPO-CNF [44,45]. The different cell culture medium used in this study affected the nanomechanical properties of the TEMPO-CNF material significantly, where RPMI resulted in a higher elastic modulus of the TEMPO-CNF compared with DMEM. This difference could arise from differences in composition since 50% of the Ca^2+^ in RPMI are replaced by Mg^2+^ and it also contains the reducing agent glutathione. It is known from crosslinking of alginates that different cations form hydrogels with varying mechanical properties [45]. The reasons for the difference in mechanical properties of the TEMPO-CNF-hydrogels is solely speculative at this stage, but if the differences are large enough to induce differences in cell responses, selection of growth medium could be important.

Cells will respond to roughness, porosity, pore size and microstructure of the material as well as chemical properties such as charge, valency and functional groups [46,47,48]. For the 3D printed TEMPO-CNF scaffolds used in this study, we show that breast cancer cells can be seeded on the scaffolds, the cells expand as expected (Figure 3A,B) and adopt a smaller cell size compared with cells grown in 2D cell culture dishes (Figure 3C), which is consistent with previous studies [46,47,48]. The cells adapted to the material surface, grew into cavities in the porous material structure (Figure 4B(ii)) and developed a heterogenous cell population (Figure 4B(ii,iii),C(ii,iii)) mimicking the in vivo situation. This is also consistent with previous work using de-cellularized patient derived scaffolds and 3D-printed alginate hydrogels where seeded cancer cells populate the entire material surface ([12,15] and unpublished data).

By analyzing biomarkers for tumor cell characteristics at mRNA level, 3D TEMPO-CNF scaffolds increased expression of genes related to stemness and migratory properties compared with 2D cultures (Figure 5). To functionally determine if cells derive from stem cells, transit-amplifying cells or differentiated cells, the ability of the cells to form holoclones, meroclones or paraclones, respectively, can be studied [49,50]. Increased ability of MCF7 and MDA-MB-231 breast cancer cells, cultured on scaffolds, to form holoclones supported the gene expression results and the 3D TEMPO-CNF scaffolds can be regarded to enrich for CSC (Figure 6).

Our results confirm our previous data showing that cells cultured in 3D-printed alginate hydrogel scaffolds fill out pores and cavities throughout the scaffold with a heterogenous population of cells in multilayers compared with cells grown in a monolayer with a homogenous cell population in 2D [15]. Previous studies have also shown that fine-tuning of the mesh network, and using different geometries, affect the cell phenotype [51].

## 5. Conclusions

Carboxylated CNF demonstrated good capability to be applied as ink in a micro-extrusion printer producing 3D scaffolds with micrometer-sized pores. The breast cancer cells lines MCF7, MDA-MB-231 and T47D were successfully cultured on the scaffolds and developed a cellular heterogeneity growing in multiple layers throughout the scaffolds with enhanced CSC features. The data suggests that TEMPO-CNF-based 3D printed scaffolds can be used as cancer cell culture models mimicking the in vivo situation in a more relevant way compared with conventional 2D cell culturing on plastics making them suitable for applications such as drug testing.

## Data Availability

The data presented in this study are available on request from the corresponding author.

## Supplementary Materials

The following are available online at https://www.mdpi.com/article/10.3390/bioengineering8070097/s1, Table S1. Primer sequences for qPCR.

## References

[1] Torre L.A., Bray F., Siegel R.L., Ferlay J., Lortet-Tieulent J., Jemal A. (2015). Global cancer statistics, 2012: Global Cancer Statistics, 2012. CA Cancer J. Clin..

[2] Yamada K.M., Cukierman E. (2007). Modeling Tissue Morphogenesis and Cancer in 3D. Cell.

[3] Zhang H., Wang Z.Z. (2008). Mechanisms that mediate stem cell self-renewal and differentiation. J. Cell. Biochem..

[4] Hutchinson L., Kirk R. (2011). High drug attrition rates—Where are we going wrong?. Nat. Rev. Clin. Oncol..

[5] Hay M., Thomas D.W., Craighead J.L., Economides C., Rosenthal J. (2014). Clinical development success rates for investigational drugs. Nat. Biotechnol..

[6] Qiao S.-P., Zhao Y.-F., Li C.-F., Yin Y.-B., Meng Q.-Y., Lin F.-H., Liu Y., Hou X.-L., Guo K., Chen X. (2016). An alginate-based platform for cancer stem cell research. Acta Biomater..

[7] Vlashi E., Pajonk F. (2015). Cancer stem cells, cancer cell plasticity and radiation therapy. Semin. Cancer Biol..

[8] Cavo M., Caria M., Pulsoni I., Beltrame F., Fato M., Scaglione S. (2018). A new cell-laden 3D Alginate-Matrigel hydrogel resembles human breast cancer cell malignant morphology, spread and invasion capability observed “in vivo”. Sci. Rep..

[9] Pampaloni F., Reynaud E.G., Stelzer E.H.K. (2007). The third dimension bridges the gap between cell culture and live tissue. Nat. Rev. Mol. Cell Biol..

[10] Edmondson R., Broglie J.J., Adcock A.F., Yang L. (2014). Three-Dimensional Cell Culture Systems and Their Applications in Drug Discovery and Cell-Based Biosensors. ASSAY Drug Dev. Technol..

[11] Langhans S.A. (2018). Three-Dimensional in Vitro Cell Culture Models in Drug Discovery and Drug Repositioning. Front. Pharmacol..

[12] Landberg G., Fitzpatrick P., Isakson P., Jonasson E., Karlsson J., Larsson E., Svanström A., Rafnsdottir S., Persson E., Gustafsson A. (2020). Patient-derived scaffolds uncover breast cancer promoting properties of the microenvironment. Biomaterials.

[13] Le X., Poinern G.E.J., Ali N., Berry C.M., Fawcett D. (2013). Engineering a Biocompatible Scaffold with Either Micrometre or Nanometre Scale Surface Topography for Promoting Protein Adsorption and Cellular Response. Int. J. Biomater..

[14] Huang L., Abdalla A.M., Xiao L., Yang G. (2020). Biopolymer-Based Microcarriers for Three-Dimensional Cell Culture and Engineered Tissue Formation. Int. J. Mol. Sci..

[15] Svanström A., Rosendahl J., Salerno S., Leiva M.C., Gregersson P., Berglin M., Bogestål Y., Lausmaa J., Oko A., Chinga-Carrasco G. (2021). Optimized alginate-based 3D printed scaffolds as a model of patient derived breast cancer microenvironments in drug discovery. Biomed. Mater..

[16] Caddeo S., Boffito M., Sartori S. (2017). Tissue Engineering Approaches in the Design of Healthy and Pathological in vitro Tissue Models. Front. Bioeng. Biotechnol..

[17] Basu A., Celma G., Strømme M., Ferraz N. (2018). In Vitro and in vivo Evaluation of the Wound Healing Properties of Nanofibrillated Cellulose Hydrogels. ACS Appl. Bio Mater..

[18] Rashad A., Mustafa K., Heggset E.B., Syverud K. (2017). Cytocompatibility of Wood-Derived Cellulose Nanofibril Hydrogels with Different Surface Chemistry. Biomacromolecules.

[19] Nordli H.R., Chinga-Carrasco G., Rokstad A.M., Pukstad B. (2016). Producing ultrapure wood cellulose nanofibrils and evaluating the cytotoxicity using human skin cells. Carbohydr. Polym..

[20] Mertaniemi H., Escobedo-Lucea C., Sanz-Garcia A., Gandía C., Mäkitie A., Partanen J., Ikkala O., Yliperttula M. (2016). Human stem cell decorated nanocellulose threads for biomedical applications. Biomaterials.

[21] Lou Y.-R., Kanninen L., Kuisma T., Niklander J., Noon L., Burks D., Urtti A., Yliperttula M. (2014). The Use of Nanofibrillar Cellulose Hydrogel as a Flexible Three-Dimensional Model to Culture Human Pluripotent Stem Cells. Stem Cells Dev..

[22] Hong N., Yang G.-H., Lee J., Kim G. (2018). 3D bioprinting and its in vivo applications. J. Biomed. Mater. Res. Part B Appl. Biomater..

[23] Moberg T., Sahlin K., Yao K., Geng S., Westman G., Zhou Q., Oksman K., Rigdahl M. (2017). Rheological properties of nanocellulose suspensions: Effects of fibril/particle dimensions and surface characteristics. Cellulose.

[24] Naderi A., Lindström T., Sundström J. (2014). Carboxymethylated nanofibrillated cellulose: Rheological studies. Cellulose.

[25] Iotti M., Gregersen Ø.W., Moe S., Lenes M. (2011). Rheological Studies of Microfibrillar Cellulose Water Dispersions. J. Polym. Environ..

[26] Ojansivu M., Rashad A., Ahlinder A.E., Massera J., Mishra A., Syverud K., Finne-Wistrand A., Miettinen S., Mustafa K. (2019). Wood-based nanocellulose and bioactive glass modified gelatin–alginate bioinks for 3D bioprinting of bone cells. Biofabrication.

[27] Xu C., Molino B.Z., Wang X., Cheng F., Xu W., Molino P., Bacher M., Su D., Rosenau T., Willför S. (2018). 3D printing of nanocellulose hydrogel scaffolds with tunable mechanical strength towards wound healing application. J. Mater. Chem. B.

[28] Chinga-Carrasco G., Ehman N.V., Pettersson J., Vallejos M.E., Brodin M.W., Felissia F.E., Håkansson J., Area M.C. (2018). Pulping and Pretreatment Affect the Characteristics of Bagasse Inks for Three-dimensional Printing. ACS Sustain. Chem. Eng..

[29] Markstedt K., Mantas A., Tournier I., Ávila H.M., Hägg D., Gatenholm P. (2015). 3D Bioprinting Human Chondrocytes with Nanocellulose–Alginate Bioink for Cartilage Tissue Engineering Applications. Biomacromolecules.

[30] Saito T., Nishiyama Y., Putaux J.-L., Vignon M., Isogai A. (2006). Homogeneous Suspensions of Individualized Microfibrils from TEMPO-Catalyzed Oxidation of Native Cellulose. Biomacromolecules.

[31] Liu J., Cheng F., Grénman H., Spoljaric S., Seppälä J., Eriksson J.E., Willför S., Xu C. (2016). Development of nanocellulose scaffolds with tunable structures to support 3D cell culture. Carbohydr. Polym..

[32] Chinga-Carrasco G., Kuznetsova N., Garaeva M., Leirset I., Galiullina G., Kostochko A., Syverud K. (2012). Bleached and unbleached MFC nanobarriers: Properties and hydrophobisation with hexamethyldisilazane. J. Nanopart. Res..

[33] Saito T., Isogai A. (2004). TEMPO-Mediated Oxidation of Native Cellulose. The Effect of Oxidation Conditions on Chemical and Crystal Structures of the Water-Insoluble Fractions. Biomacromolecules.

[34] Silva F., Gracia N., McDonagh B.H., Domingues F.C., Nerín C., Chinga-Carrasco G. (2019). Antimicrobial activity of biocomposite films containing cellulose nanofibrils and ethyl lauroyl arginate. J. Mater. Sci..

[35] Espinosa E., Filgueira D., Rodríguez A., Chinga-Carrasco G. (2019). Nanocellulose-Based Inks—Effect of Alginate Content on the Water Absorption of 3D Printed Constructs. Bioengineering.

[36] Bustin S.A., Benes V., Garson J.A., Hellemans J., Huggett J., Kubista M., Mueller R., Nolan T., Pfaffl M.W., Shipley G.L. (2009). The MIQE Guidelines: Minimum Information for Publication of Quantitative Real-Time PCR Experiments. Clin. Chem..

[37] Schneider C.A., Rasband W.S., Eliceiri K.W. (2012). NIH Image to ImageJ: 25 years of image analysis. Nat. Methods.

[38] Webb B., Doyle B.J. (2017). Parameter optimization for 3D bioprinting of hydrogels. Bioprinting.

[39] Jose R.R., Rodriguez M.J., Dixon T.A., Omenetto F., Kaplan D.L. (2016). Evolution of Bioinks and Additive Manufacturing Technologies for 3D Bioprinting. ACS Biomater. Sci. Eng..

[40] Nordli H.R., Pukstad B., Chinga-Carrasco G., Rokstad A.M. (2019). Ultrapure Wood Nanocellulose—Assessments of Coagulation and Initial Inflammation Potential. ACS Appl. Bio Mater..

[41] Liu J., Chinga-Carrasco G., Cheng F., Xu W., Willför S., Syverud K., Xu C. (2016). Hemicellulose-reinforced nanocellulose hydrogels for wound healing application. Cellulose.

[42] Radisic M., Alsberg E. (2017). Special Issue on Tissue Engineering. ACS Biomater. Sci. Eng..

[43] Butcher D.T., Alliston T., Weaver V.M. (2009). A tense situation: Forcing tumour progression. Nat. Rev. Cancer.

[44] Engler A., Sen S., Sweeney H.L., Discher D.E. (2006). Matrix Elasticity Directs Stem Cell Lineage Specification. Cell.

[45] Loh Q.L., Choong C. (2013). Three-Dimensional Scaffolds for Tissue Engineering Applications: Role of Porosity and Pore Size. Tissue Eng. Part B Rev..

[46] Mrksich M., Chen C., Xia Y., Dike L.E., Ingber D.E., Whitesides G.M. (1996). Controlling cell attachment on contoured surfaces with self-assembled monolayers of alkanethiolates on gold. Proc. Natl. Acad. Sci. USA.

[47] Arima Y., Iwata H. (2007). Effects of surface functional groups on protein adsorption and subsequent cell adhesion using self-assembled monolayers. J. Mater. Chem..

[48] Lee M.H., Brass D.A., Morris R., Composto R.J., Ducheyne P. (2005). The effect of non-specific interactions on cellular adhesion using model surfaces. Biomaterials.

[49] Pellegrini G., Golisano O., Paterna P., Lambiase A., Bonini S., Rama P., De Luca M. (1999). Location and Clonal Analysis of Stem Cells and Their Differentiated Progeny in the Human Ocular Surface. J. Cell Biol..

[50] Barrandon Y., Green H. (1987). Three clonal types of keratinocyte with different capacities for multiplication. Proc. Natl. Acad. Sci. USA.

[51] Callens S.J., Uyttendaele R.J., Fratila-Apachitei L.E., Zadpoor A.A. (2020). Substrate curvature as a cue to guide spatiotemporal cell and tissue organization. Biomaterials.

