# Supplementary material for: 3D Printed Nanocellulose Scaffolds as a Cancer Cell Culture Model System
Source: Bioengineering (Basel). 2021 Jul 10;8(7):97. doi: 10.3390/bioengineering8070097 (PMC8301137; doi:10.3390/bioengineering8070097)
Supplement: Supplementary file 1 [file bioengineering-08-00097-s001.zip › bioengineering-1240854-Supplementary Materials.pdf]

Table S1. Primer sequences for qPCR.

	Gene Name	Forward Primer	Reverse Primer
Breast cancer stem cell	*CD44*	GAAGAAGGTGTGGGCAGAAGA	ACCATTTCCTGAGACTTGCTG
EMT	*TWIST1*	GGACAGTGATTCCCAGACGG	CATAGTGATGCCTTTCCTTTCAG
	SNAI1	TAATCCAGAGTTTACCTTCCAGCA	AGCCTTTCCCACTGTCCTCA
	MUC1	CTGGTCTGTGTTCTGGTTGC	CCACTGCTGGGTTTGTGTAA
	VIM2	CAGATGCGTGAAATGGAAGA	TGGAAGAGGCAGAGAAATCC
Pluripotency	*POU5F1*	CGAAAGAGAAAGCGAACCAG	AACCACACTCGGACCACATC
	SOX2	ACACCAATCCCATCCACACT	CCTCCCCAGGTTTTCTCTGT
	NANOG	CCTATGCCTGTGATTTGTGG	AAGTGGGTTGTTTGCCTTTG
Proliferation	*MKI67*	TGGGTCTGTTATTGATGAGCC	CATCAGGGTCAGAAGAGAAGC
	CCNA2	AAGACGAGACGGGTTGC	GGCTGTTTACTGTTTGCTTTCC
	ERBB2	ACCTGGAACTCACCTACCTG	TCACTTGGTTGTGAGCGATG
Differentiation	*CDH1*	AGAGGACCAGGACTTTGACTTG	CAGAGAATCATAAGGCGGGG
	PGR	TAAATGAACAGCGGATGAAAGAA	CGACACAACTCCTTTTTGCCT
	EPCAM1	CAGGAAGAATGTGTCTGTGAAAACT	TTCATTTCTGCCTTCATCACC
	CD24	GCTCCTACCCACGCAGATT	GGTGGTGGCATTAGTTGGAT
Reference genes	*EIF1*	TCGTATGTCCGCTATCCAGA	TAAGGGTCTTCCTGCCGTTT
	YWHA2	ACGCCTCACTCCCGTTT	CTGGATGTTCTGCTGGCTC
	GAPDH	AGTCAGCCGCATCTTCTTTT	CGCCCAATACGACCAAAT
	RPS10	AGCCGCAGAGATGTTGATG	CCTCGGGACTTGAGAGACTG
	RPS26	GATGCGTGCCCAAGGAC	CAGGTCTAAATCGGGGTGG
